# Retraction Note: miR-340-FHL2 axis inhibits cell growth and metastasis in ovarian cancer

**DOI:** 10.1038/s41419-026-08454-1

**Published:** 2026-02-05

**Authors:** Zheng Huang, Qiuxia Li, Kaili Luo, Qinkai Zhang, Jingwen Geng, Xunzhu Zhou, Yesha Xu, Mengyao Qian, Jian-an Zhang, Liying Ji, Jianmin Wu

**Affiliations:** 1 Institute of Genomic Medicine, Wenzhou Medical University, 325000 Wenzhou, Zhejiang P. R. China; 2 Department of Obstetrics and Gynecology, The Second Affiliated Hospital and Yuying Children of Wenzhou Medical University, 325027 Wenzhou, Zhejiang P. R. China

Retraction Note to: *Cell Death & Disease* 10.1038/s41419-019-1604-3, published online 08 May 2019

The Editors have retracted this article. After publication, concerns were raised regarding the data presented in the figures, specifically:

- Fig. 2e bottom right image appears highly similar to that in the authors' later article [1];
- Fig 4a A2780 LV-miR-NC appears to overlap with Fig. S4d A2780 siCON (with rotation);
- Fig. S4d A2780 siFHL2-1 and siFHL2-2 appear to overlap;
- Fig. 7c SKOV3 p-b-catenin blot appears highly similar to Fig. 7e p-b-catenin lanes 1 and 2.

The Editors therefore no longer have confidence in the presented data.

Jianmin Wu does not agree with this retraction. The other authors have not responded to any correspondence from the editor or publisher about this retraction.
